# Assessing Knowledge and Attitudes of Diabetes in Zuni Indians Using a Culture-Centered Approach

**DOI:** 10.1371/journal.pone.0099614

**Published:** 2014-06-11

**Authors:** Sara Newman, Terri Cheng, Donica M. Ghahate, Jeanette Bobelu, Phillip Sandy, Thomas Faber, Vallabh O. Shah

**Affiliations:** 1 Health and Behavioral Sciences, University of Colorado, Denver, Colorado, United States of America; 2 School of Medicine, University of New Mexico Health Sciences Center, Albuquerque, New Mexico, United States of America; 3 Indian Health Services Comprehensive Center in Zuni Pueblo, Zuni, New Mexico, United States of America; Iran University of Medical Sciences, Iran, Islamic Republic Of

## Abstract

**Introduction:**

The Zuni Pueblo, in collaboration with the University of New Mexico, have formed the Zuni Health Initiative (ZHI) engaged in community-based participatory research to plan and implement educational interventions to reduce health disparities. We conducted the first phase of ZHI study and identified barriers to healthcare. We concluded that the burden presented by these barriers ultimately translates into a lack of patient activation and engagement in their health care including for diabetes, effectively hindering adoption of healthy behaviors.

**Methods:**

Community health representatives (CHRs) led 10 one-hour focus group sessions to elicit information on diabetes knowledge and self-management strategies at which a total of 84 people participated. Audiotapes were translated and transcribed by bilingual ZHI staff. We reduced the text to thematic categories, constructed a coding dictionary and inserted the text into NVivo 9 program.

**Results:**

The focus groups revealed that despite extensive personal or family experiences with diabetes or complications, participants identified knowledge gaps in the disease progression and disease management. However, we gained insight into how many Zunis conceptualize the etiology of diabetes, risk factors associated with diabetes, sources of knowledge and self-management practices.

**Conclusion:**

We concluded that many of the Zuni diabetics experience significant impacts on their life when they were diagnosed with diabetes and suffered the plight of stigmatization. We further concluded that developing Zuni culture specific diabetes care should focus on family involvement with continued education.

## Introduction

Health disparities are driven by demographics, economics, geography, cultural beliefs and a distrust of established healthcare delivery models. For generations, the American Indians/Alaska Natives (AI/AN) accepted way of life favored balance in body, mind, and spirit. However, due to the displacement, cultural/historical trauma, and high rates of poverty have taken a heavy toll on Native peoples and their ways of life with 1 in 5 now has two or more chronic health problems. The Indian Health Service (IHS) provides limited health care to AI/AN. Yet more than 4 in 10 AI/AN have no access to IHS health service.

### Epidemic of Diabetes

Diabetes has reached epidemic proportions throughout the United States, placing a significant burden on health and economics in the United States. The National Health and Nutrition Examination Survey (NHANES) have estimated the prevalence of diagnosed and undiagnosed diabetes to be about 25.8 million individuals or 8.3% of the population in 2010 [1]. Diabetes has estimated direct and indirect medical costs exceeding $245 billion in the United States in 2012 [1]. The rates of diabetes and severity of complications from diabetes is significantly greater in Native American populations than in any other population in the United States. Data from the NHANES and the Indian Health Service’s National Patient Information Reporting System indicates that 14.2% of the America Indian adults receiving care from Indian Health Services are diabetic with an adjusted rate of 16.1% of the total adult American Indian population [1].

### Zuni Indian Context

The Zuni Pueblo, located in a rural region of western New Mexico, has a population of about 11,000 Zuni Indians. Low rates of emigration and immigration have resulted in an endogamous population characterized by large multigenerational families. The median age in the Zuni Pueblo is 26 years, and the major source of income is through home-based jewelry and fetish making [2]. Changing lifestyles and systemic forces have led to decreased physical activity and increased caloric intake through consumption of calorie-dense foods. These factors have created a socio-economically disadvantaged population that is a major public health challenge due to growing health disparities that have resulted in interrelated epidemics of obesity, diabetes, hypertension, and kidney disease. In addition, decreasing the impact of these epidemics is complicated by a unique combination of historical, economic and cultural barriers, which limit health care utilization thereby increasing health disparities [2]–[3].

Addressing health disparities in the Zuni begins with consideration of the “fourth world” context of American Indians, referring to the existence of an indigenous minority where institutionalized power and privilege are held by a colonizing, subordinating majority [4]. The cumulative effect of the subordination of American Indians has been a “soul wound” which reflects historical trauma. As such, reducing heath disparity among the Zuni requires a new approach that recognizes the impact of these historical injustices. In addressing obesity and diabetes in the Zuni population, it is necessary to plan intervention strategies that are consistent with the local traditional values and knowledge. Collecting input from tribal community members can provide the direction for these types of interventions. As seen with other successful community-based strategies, there needs to be an emphasis on community partnership and cultural perspective.

### Theoretical Framework: Identifying Cultural Barriers to Health Care in Zuni

The particular culture-centered knowledge regarding the process of diabetes, health behaviors and nutrition are unknown in the specific Zuni context. Contextual factors are especially important when examining health behaviors, as human experience becomes more meaningful when articulated within the context in which it is experienced [5]. This theoretical foundation informs the study approach that places community interests at the core of the definition of the problem as well as the development of solutions to problems [5]. In order to address the need for a contextualized or culturally informed understanding of knowledge we engaged in a qualitative study designed to probe the specific culture-bound knowledge and understanding Zuni Indians have about the disease process of type 2 diabetes mellitus (T2DM) and the effect of health behaviors and nutrition [6]. Focus group participants were asked to respond to the following Zuni culturally specific questions about diabetes: “What causes diabetes?; Who is at risk for getting diabetes?; Asking about how the doctor explained diabetes and what it means to the patient; how can a diabetic avoid symptoms of diabetes; what are the barriers in managing diabetes; who helps most in managing diabetes; what programs are needed in the community to help manage diabetes.” Probes and follow-up questions were used to explore dominant themes and expand upon the topics raised during the discussion. Many of the participants responded by speaking partly in the Zuni language, “Shiwi,” mixed with English. Participants also completed a CHR-administered survey questionnaire, which provided demographic, medical history, environmental exposure, family history, and physical assessment information. These questions are summarized in table 1. The objective was to uncover the various ways of understanding and negotiating the meaning of diabetes embedded within the Zuni specific cultural context [5]. Due to this, we used a focus group approach, which attempts to look at group level processing as well as habitual behaviors that are best addressed through focus group methodology [7]. Through using focus groups several themes emerged including the following: (1) cultural knowledge of T2DM, (2) sources knowledge of T2DM (3) risk factors for developing T2DM (4) self-management techniques of T2DM. Using this data, educational resources specific to the Zuni population will be developed and incorporated in interventions that combine the individual, family, community, and IHS to slow the rate of increase in T2DM.

**Table 1:** Focus Group Guide.

Category	Questions:
Definition	What is diabetes? (Or what do you know about diabetes?)
Diabetes etiology	a. What causes diabetes? (Probe: Ask about sugar, carbs, fat, exercise, genetics)
	b. Who is at risk for getting diabetes? (Probe: Are Zunis at risk because of food, lifestyle, genes, and family history?)
Patient response to diabetes diagnosis	Please tell the story of how you or your family member found out you or they had diabetes. a. How did the doctor explain diabetes to you? What did s/he tell you about the disease? What did you think it would mean to have diabetes? (Probe)
	b. How did you feel when you found out you had diabetes? (Probe)
Self-management	What can you do to avoid the symptoms of diabetes and to keep your diabetes from getting worse? (Probe: Query about medications, side effects, compliance in lifestyle and medications)
Barriers to self-management	What prevents you from managing your diabetes?
Family participation in diabetes management	a. Who helps you the most with your diabetes?
	b. How does this person help you?
Management programs	What programs would you like to see in your community to help you manage your diabetes?
Questions used for probing	1. How do you and your family eat?
	2. What is healthy food?
	3. What is not healthy?
	4. What role do you think diet has on the development of diabetes?
	5. What do you know as traditional food and is it healthy?
	6. What could we do to motivate our people to eat more of what you said is healthy?
	7. Do you think you’ve learned something?

## Methods

This study was approved by the University of New Mexico Health Sciences Center Human Research Review Committee and the Indian Health Service Institutional Review Board.

### Recruitment

Initially, potential study participants were recruited by Zuni community health representatives (CHRs) using the Zuni Health Initiative (ZHI) project’s clinical database containing records on 25% members of the Zuni community. Individuals were recruited through visits by CHRs to Zuni households; presentations at tribal health programs and at health care centers, distribution of flyers at local businesses and civic centers, and through other health programs. Overall we approached about 100 households and recruited individuals (n = 84) to participate in 10 one-hour focus group discussions. Individuals were required to either (1) have a diagnosis of diabetes or prediabetes, based on their hemoglobin A1C level; or (2) be a caretaker of a family member with diabetes. Recruitment was not intended to ensure a randomly selected sample of respondents but by using a variety of recruitment strategies and recruiting a large sample size, it was assumed diverse viewpoints were represented in the focus groups. The minimum numbers of participants in each focus group were not less than 7 and maximum numbers of participants were not more than 11.

### Description of Focus Group Sessions

The focus group sessions were conducted at the ZHI office between May through August 2012. One researcher or CHR facilitated each focus group session. Zuni community members with a background in health-related work were recruited, trained and certified as lay interventionists by the University of New Mexico’s Project Extension for Community Healthcare Outcomes (Project ECHO). They were trained on qualitative focus group, lifestyle coaching, diabetes prevention, and diet and exercise change. The CHR staff has also received extensive training in adult education including i) diabetes management, ii) the theoretical framework of intervention, iii) group management skills, and iv) implementing an interventional protocol. The initial training was approximately 100 hours in duration and was performed using 30 hours of face to face and rest as WEB-education. Dr Deborah Helitzer, Sc.D. at University of New Mexico is an expert in qualitative research who conducted face to face teaching with the Zuni staff about the logistic of focus groups and certified all Zuni CHR’s after monitoring couple of focus group session conducted by CHRs. Each focus group session was tape-recorded. In addition, two more CHRs were present during each session to record notes during the discussion. A focus group guide was developed by the principal investigator (VOS) in collaboration with Zuni I H S diabetes clinic. Questions are listed in table 1. The guide contained an introduction, group guidelines that assured confidentiality, and a series of questions about knowledge and personal experiences with diabetes developed from a literature review. Participants completed a CHR-administered survey, which provided demographic, medical history, environmental exposure, family history, and physical assessment information. They also had clinical laboratory drawn to evaluate their hemoglobin A1C, renal function, and lipid profile. We administered and received original, written and signed Institutional Review Board approved consent and Health Insurance Portability and Accountability Act forms from all participants and participants received $25 for participation.

### Data Collection and Analysis

We used a systematic text-analysis procedure to ensure high quality data. Trained Zuni-speaking ZHI employed staff transcribed the audiotapes of the focus groups immediately after the event. Staff members used their verbatim notes taken during the focus group sessions to facilitate transcription. The ZHI office has all required resources including clinical office and recording, computing and electronic devices to conduct transcription and translation of focus group recording which took about 6–8 hours per each focus group. Transcripts were analyzed using a grounded theory approach, allowing us to create conceptual frameworks and theories based on inductive analysis of the data in order to identify participants’ main concerns [8].

Systematic reading and coding of the transcripts were done by two of the authors (VOS, and SN). The transcripts from individual focus groups were reviewed to identify themes and categories using an inductive approach [9]. This process was used to develop categories, which were used to create the themes found. Interview text was coded into QSR International’s NVivo 9 qualitative data analysis software (http://www.qsrinternational.com/support_faqs_detail.aspx?view=11). Memos of the emergent analysis of larger themes were written, incorporating quotes from the text to support findings.

The quantitative demographic, medical history, environmental exposure, family history, physical assessment information, and lab results were entered into SAS version 9.2 and descriptive statistical analysis was conducted. The percentages for the categorical items are shown (Table 2).

**Table 2:** Focus group participant characteristics (n = 84).

Demographic	Percent (%)	Clinical Characteristics	Percent (%)
**Gender**			
Female	58	Type 2 Diabetes	64
**Age group (years)**			
18–25	8	Hypertension	54
26–35	18	Hypertension and diabetes	41
36–45	33		
46–55	24	Overweight (BMI 25–29.9)	32
56–65	13	Obese (BMI >30)	49
65+	4		
**Education**		Total Cholesterol	19
Less than high school	36		
High School diploma	32	Microalbuminuria(UACR30–300)	34
Beyond High school	32	Albuminuria (UACR >300)	14
**Artisan**	42	Diabetes and Kidney Disease	22

## Results

### Participants

Demographic characteristics are summarized in Table 2. Of the 84 participants, 58% were women and most participants were over the age of 26 years and the age range for the focus group was from 21 years to anyone who was living in the Zuni community. 64% of participants had a diagnosis of diabetes as reported in the survey questionnaire. Of those with diabetes, 22% reported diabetic kidney disease. The clinical measurements of A1C data showed that 42% were considered non-diabetic or had well-controlled diabetes mellitus (DM); 22% had an A1C of 5.7–6.5% putting them in the pre-diabetic range, and 36% had an A1C >6.5%. Hypertension was reported by 53% of participants, while 34% of participants had microalbuminuria and 14% had albuminuria, indicating presence of kidney disease. The majority of patients, 80% were considered overweight or obese, based on a Body Mass Index >25. Participants reported being artisans were 42%. About 32% of participants reported education beyond high school. In addition, about 32% of participants had received less than 12 years of education. Approximately half of the participants responded by speaking in the Zuni language, “Shiwi”, mixed with English, which was translated by CHRs.

### Knowledge of Diabetes

All participants experience significant impacts of diabetes diagnosis on their lives with common description of the trouble of stigmatization within family and tribe. When asked specifically about the definition of diabetes, participants viewed diabetes through organ-specific as well as domain-specific frameworks. Organs identified were the kidney or pancreas and domains were framed through sugar or insulin. Several participants described pancreas dysfunction as the main cause. Others associated diabetes with sugar intake and improper insulin productions.

One participant defined diabetes as,

*“When your body can’t really make that excess sugar, you can’t burn it all and it just like sticks. Sticks in one place so therefore, when it all builds, it, it can’t burn sugar quite as a normal person could.”*

Other participants described symptoms of the disease, in order to define it. Instead of relating clinical knowledge, they expressed the lived experience of managing diabetes symptoms. Several participants mentioned fear of blurry vision, dizziness, and diabetic coma. One participant described,

*“When your sugar gets high, you can feel like going to the bathroom frequently and drinking too much water, you get dizzy spells, makes you weak, and you forget to take your medication, and you get dehydrated.”*

Participants frequently mentioned the kidney and other physical complications when asked to describe diabetes. Several mentioned kidney disease and dialysis as concerns to the community as well as concerns of eye sight degradation and amputation. Others shared stories of complications faced by friends and family members. One participant described his experience of diabetes complications with his family, *“my mother died from congestive heart failure from that, there’s amputations that can come from that too, blindness”.*

### Sources of Knowledge

Participants reported several sources of knowledge about diabetes including family, friends, and healthcare workers. Several participants described receiving diabetes information from friends and family but not a health professional. Other participants were diagnosed with diabetes and educated by health professionals. One participant spoke about taking advantage of the time she had with her provider to gain more knowledge on diabetes:

*“So a big thing too is that when you go see a doctor or health provider, I just barely changed my mentality on that too. Yeah it’s your time ask those questions, you’re tired of it and waited too long, so when is another time you’re gonna see this doctor again so take advantage of that. So that’s just all this took a long while to set my mentality straight on that.”*

Other participants went to other providers such as dieticians or psychiatrists to get assistance in managing diabetes. One participant said of these providers, *“They teach you…Just anything that you want to take in, they will tell you. Then they’ll show you how to use the monitor.”* Participants reported receiving much of their information from community members and diabetes educators but did not seem to utilize their physicians as much, resulting in a large variation in the information they received.

### Knowledge of Risk Factors for Diabetes

A common theme that emerged in the focus groups was the idea that a large proportion of American Indians have diabetes. Participants mentioned other tribes outside of Zuni as facing large rates of diabetes, but with a special focus on the Zuni tribe. This participant said, “it’s kind of sad because you know Zunis I heard that Zunis the major tribe in the U.S. that is affected.”

Respondents talked of diabetes as passed down through generations. Participants viewed diabetes as inevitable when multiple family members were diagnosed with diabetes. Others found genetics to be of more importance than lifestyle factors such as diet and exercise. One participant viewed it in terms of risk:

*“you’re high risk that you’re gonna get it or if it runs in the family. For me, it’s both sides of my parent that has it and that’s how I got it; I was at a high risk.”*

Participants attributed the high rates of diabetes in Zuni people to a large change in lifestyle related to diet and activity level from past generations of Zunis. Respondents identified a decrease in physical activity from no longer farming as well as a change in diet from growing and hunting food to eat. One response was:

*“Our ancestors used to farm and were very active they ate what they grew and they ate what they hunted, but as society grew and came into our community and there started coming in processed food.”*

Participants identified diet as a major cause of diabetes as well as obesity. Several participants mentioned portion size, and specific food culprits such as lard and sugar. Participants were also concerned with the amount of sugar ingested, “*Especially I see a lot of people drinking soda and not drinking water. And that’s just sugar, just adds up to your body.*”

Exercise was also identified an important factor in the lifestyle discussion. A participant said, “*You can get diabetes with lack of exercise.”* Inactivity attributed to people’s work in Zuni was a common theme mentioned.

*“The sedentary lifestyle in Zuni, they do silver work, carvings, or whatever and most of the time it’s spent sitting down, not really moving at all and that you just eat, flop back down in front of your work table and work and just wait till, till the next meal.”*

Alcohol abuse was another theme that emerged when asked about causes of diabetes. Several participants mentioned alcohol as a direct cause of diabetes,

*Another participant talked about the mechanism in which alcohol affects the body, “Also you can provoke it more through like through like abusing your body with alcohol because it affects the pancreas quicker. It’ll ah, alcohol goes through the body and it tries to break it down but all that sugar comes out of it.”*

Other participants found that alcohol abuse contributed to poor diabetes management. There were different ideas to the cause, some identified alcohol abuse as the cause of poor management; others viewed alcohol abuse as a way to cope with a diabetes diagnosis.

### Knowledge of Self-management Methods

Participants reported a variety of methods in ways to prevent or manage diabetes. The main themes that emerged were exercise, eating strategies, education, medications, and taking advantage of diabetes programs offered in the community. Exercise was mentioned frequently as a way to prevent or manage diabetes. One participant said, *“Well I do exercise every week. I come up here well, I try to come up here every week or every day…but I love exercising because I don’t think I’ll have diabetes.”*

Participants shared strategies for healthier diets in order to manage their diabetes. One strategy was to limit unhealthy foods, which were defined as packaged foods, sugary snacks, fatty meats, and added salt, and eat healthier foods like fresh fruits and vegetables. Other participants described controlling portion sizes, “*It’s not what you eat it’s how much you eat, and you have to have 3 meals a day but small portions.*”

Education was also identified as a way to prevent or manage diabetes. One participant suggested being more active about understanding blood results, “*Learn about your A1C. That’s a good one to learn, it measures your blood sugar for the last 3 months.*” Another participant agreed and described tracking her blood sugar with a glucometer, “*Watching your sugar levels you know there’s a meter you can use, which is good. It tells you if you’re okay or if your sugar levels are high or normal or sometimes it might be low but I control it.*”

Several participants spoke of managing their diabetes with medication. Some participants found that managing their diabetes with medication is working, while others were reluctant to take medications and expressed desire to decrease or stop them. One participant described taking medication as a way of managing “*some kind of poison in your system*.” Several participants were scared of getting to an advanced disease state where they would require daily insulin shots.

Other participants on medications for their diabetes voiced that they would prefer to stop them. One participant was distrustful of medications, viewing them as harmful for the body, “*When they find out you’re a diabetic, they start you on medications to where it’s not right for your body. That’s why I don’t want to take any medications for that. I can do exercises on my own, lose weight and take care of myself*.”

While participants had direct knowledge of diabetes, several were confused about risk factors including lifestyle and hereditable factors. Questions included, “*With diabetes, is that like, if you’re overweight, you can get it*?” Also, *“I was wondering if it was because of too much cholesterol or sugar intake and not taking care of you more or less.”* Confusion over whether diabetes was heritable was also mentioned,

*“We were wondering too if it’s is hereditary. I don’t know if it’s well like they say it’s linked to kidneys or whatever and I have a kidney problem on my father’s side.”*

## Discussion

This focus group study demonstrated how Zunis with T2DM conceptualize their diabetic status. Despite the prevalence of diabetes and having personal experiences with diabetes, participants identified knowledge gaps in the definitions of diabetes, disease progression and disease management. They also reported social stigma and taboos as a barrier to diabetes self-management. Stresses from these experiences have shaped the Zuni people’s definition and views of diabetes to predominantly include end organ disease complications like kidney failure or blindness. In addition, we found that participants believed the progression of diabetes was inevitable in American Indian communities. Participants felt T2DM to be a serious problem in their community, identifying it as an illness nearly all American Indians faced.

As asserted in previous literature, cultural factors need to be considered in the care of people with diabetes [10]–[14] and in modifying diabetes education programs to determine patients’ knowledge and reading level and in using cultural themes to deliver health messages [10], [15]–[16]. The results in this study further support previous recommendations for the use of community engagement, as described in the CTSA manual “Principles of Community Engagement” (2011), to engage the stakeholders in a mutually responsive and respectful partnership. A major component of this approach seeks to situate the location of expertise both with community members as well as researchers and clinicians [5]. Through respecting values of local culture and the construction of programs that seek to incorporate both culture-bound knowledge as well as clinical knowledge, we may likely see more effective diabetes prevention and management [17]. Many of the AI/ANs and in particular Zuni Indians still favor their own healing practices (e.g. visiting with medicine men and other tribal healers) in contradiction to our current evidence-based medicine. Sadly such practices do not address the core issues about the medication adherence, lifestyle changes and nutrition leading to complications of T2DM including renal failure [18]–[20]. The true link between culture and health beliefs and behavior is notably valued in the treatment of diabetes involving the patterns of eating, physical activity and culturally rooted behaviors. If diabetes treatment recommendations are to be effective, they must be sensitive and relevant to the culture of the people who are expected to carry them out. Therapeutic approaches to enhance resiliency can also supplement diabetes education to help with positive coping strategies, improved attitudes about living with diabetes, and improved diet and exercise habits [21]–[23].

Results of this study supports the identified needs for additional resources focusing on culturally-centered educational materials and coordinated services to address emotional and health-related barriers [24], In order to alleviate some of the stress and develop effective educational programs, the incorporation of community health workers should be continued. Involvement of community health workers in diabetes programs has been previously studied in several minority populations and has demonstrated improvements in participant knowledge and behavior [24].

## Conclusion

In summary, we collaborated with Zuni community members to evaluate the culturally-centered knowledge that contribute to the prevalence of poor diabetes management and disease progression experienced in Zuni. Our focus group revealed that people with diabetes experience significant impacts on their life including stigmatization. We further learned about ideas from the community to develop a culturally sensitive model to address diabetes care. From this information, we concluded that developing Zuni culture specific diabetes care should focus on family involvement with continued education. We hope to further address health promotion and disease prevention through collaborative efforts between community members, healthcare workers, and local policy makers to prioritize resources for the prevention and management of diabetes in Zuni in order to reduce health disparities.
